# Immune Dysregulation in Depression and Anxiety: A Review of the Immune Response in Disease and Treatment

**DOI:** 10.3390/cells14080607

**Published:** 2025-04-17

**Authors:** Christopher Hole, Akash Dhamsania, Cassandra Brown, Rebecca Ryznar

**Affiliations:** 1 College of Osteopathic Medicine, Rocky Vista University, Englewood, CO 80112, USA; christopher.hole@co.rvu.edu (C.H.); akash.dhamsania@co.rvu.edu (A.D.); rryznar@rvu.edu (R.R.); 2 Department of Biomedical Sciences, Rocky Vista University, Englewood, CO 80112, USA

**Keywords:** depression, anxiety, inflammation, immune system, cytokine

## Abstract

Rates of depression and anxiety have increased significantly in recent decades, with many patients experiencing treatment-resistant symptoms. Beyond psychiatric manifestations, these conditions are associated with heightened risks of suicide, cardiovascular disease, chronic pain, and fatigue. Emerging research suggests that neuroinflammation, immune dysregulation, and hypothalamic–pituitary–adrenal axis dysfunction contribute to their pathophysiology, often interacting bidirectionally with stress. While current first-line treatments primarily target neurotransmitter imbalances, many patients do not achieve symptom resolution, highlighting the need for novel approaches. This review explores the role of immune dysfunction, cytokine activity, and neurotransmitter interactions in depression and anxiety. Additionally, we examine how existing pharmacological and non-pharmacological interventions influence inflammation and immune responses. Understanding these mechanisms may pave the way for more integrative treatment strategies that combine immune modulation with traditional psychiatric therapies.

## 1. Introduction

The prevalence of depression and anxiety has steadily increased since the early 2000s, with a significant rise during the global COVID-19 pandemic [1,2]. Notably, nearly 30% of patients with depression and 50% of those with generalized anxiety disorder meet the criteria for treatment resistance and may not respond to first-line interventions [3,4]. Given the increasing prevalence and the challenge of finding adequate therapeutic responses, it is essential for healthcare providers to consider the multifactorial nature of these disorders.

While depression and anxiety are commonly recognized by their hallmark psychiatric symptoms, such as loss of interest, sleep disturbances, fatigue, and general unease, it is increasingly understood that these conditions also involve disruptions in immune system regulation, neurotransmitter balance, and inflammatory activity [5,6,7]. Expanding our understanding of these disruptions could shed light on why certain patients respond better to treatments and pave the way for novel therapeutic approaches. In this discussion, we explore the role of inflammation and its effects on the brain’s protective barriers, as well as the relationships between immune dysregulation, neurotransmitter imbalances, and the pathophysiology of depression and anxiety. Additionally, we review the anti-inflammatory properties of commonly prescribed antidepressants and anxiolytics, along with potential over-the-counter therapies—such as herbal supplements—that have shown promise in managing these conditions.

## 2. Inflammation

Inflammation results from an activated immune system, involving both innate and adaptive cellular responses. During this process, immune cells and tissues release proteins and signaling compounds that trigger local and systemic responses throughout the body. The key features of these responses include increased temperature, vascular permeability, cell chemotaxis, and further activation of immune cells, which in turn release effector compounds. Inflammation persists until the initiating factor is removed, and pro-inflammatory signaling decreases. The cessation of inflammation occurs through a reduction in pro-inflammatory signals and an active shift toward anti-inflammatory signaling, which takes over after the initial phase [8].

Inflammation can arise from various sources, broadly categorized as exogenous or endogenous. Exogenous inflammation is triggered by external signals, such as allergens, irritants, and microbes, whereas endogenous inflammation is seen in autoimmunity, cancer, and stress-induced immune responses. In these cases, the immune system erroneously targets the body’s own tissues [9]. Acute inflammation is a protective response to infections or tissue damage, necessary for removing harmful agents and repairing the body. However, chronic inflammation arises when these signals persist without resolution, leading to prolonged tissue damage and increased risks for conditions like cardiovascular disease, diabetes, and psychiatric disorders such as depression and anxiety [10,11,12].

Cytokines are signaling molecules released by immune cells that regulate inflammation. Pro-inflammatory cytokines, such as IL-1β, IL-6, and TNF-α, play a key role in sustaining inflammation, while anti-inflammatory cytokines, like IL-4, IL-10, and TGF-β, help resolve the inflammatory process (Table 1). The balance between these pro- and anti-inflammatory cytokines determines the overall immune response [13]. While inflammation can cause damage throughout the body, its effects on the brain are particularly significant for understanding neuroinflammatory disorders.

### Inflammation, Immune Cells, and Brain Health

The blood–brain barrier (BBB) is a protective system that isolates the brain from the outer environment, consisting of endothelial cells, pericytes, glycocalyces, smooth muscle cells, and glial cells [14,15]. These structures form tight junctions and electromagnetic forces that regulate the internal environment through transporters and aquaporins, while antioxidant enzymes protect against harmful oxidizing compounds [16]. Selective regulation is essential to maintaining the integrity of central nervous system (CNS) functions.

Despite its protective layers, the BBB can be disrupted during pathological states, such as inflammation, which breaks down tight junctions and alters basement membrane composition [14]. While some level of cytokine signaling is necessary for brain development and synaptic management, excessive inflammation can disrupt the CNS environment, compromising BBB integrity [17]. In preclinical models, sepsis-induced degradation of the extracellular matrix is mediated by metalloproteinase activity triggered by TNF-α and IL-1β. Astrocyte activation also downregulates tight junction components via VEGF-A secretion, while microglial activation releases inflammatory mediators that increase vascular permeability [16,18]. Chronic inflammation can lead to a cumulative effect, eventually overcoming the BBB’s protective capacity [15].

Similar mechanisms of BBB disruption observed in animal and cellular models are believed to occur in humans as well. A 2021 review of brain endothelial cell responses to inflammation revealed changes in the permeability of human tissue samples similar to those observed in murine models [18]. Recent studies using dynamic contrast-enhanced MRI have linked COVID-19 infection with brain fog and BBB dysfunction in human patients, with the hypothesis that the massive systemic inflammation induced by severe infection may be responsible [19]. However, more research with larger sample sizes is needed to expand on this emerging concept.

Preclinical cell studies have supported the concept of a neuro-immune axis [20]. Immune cells communicate through autocrine and paracrine signaling, most notably with acetylcholine, dopamine, serotonin, noradrenaline, glutamate, GABA, and substance P. The microglial response also varies between neurotransmitters, local tissue environments, and receptor types (Table 2). The downstream effects of excitatory stimulation include activation of immune cells, release of cytokines, and immune cell migration. Inhibitory stimulation can result in decreased transcription of pro-inflammatory molecules, cellular degranulation, and cell maturation [21]. This neuro-immune interaction suggests bidirectional crosstalk between the immune and nervous systems, which may contribute to psychiatric disorders such as depression and anxiety.

The concept of “sickness behavior”—the link between immune activation and behavioral changes—was first observed in animal models and has since expanded to include neuroinflammatory processes. Cytokine-induced immune activation, particularly via IL-1, can alter neurotransmitter signaling in the brain, leading to changes in mood and behavior [25]. This interaction supports the notion of a neuro-immune axis that plays a role in psychiatric illnesses, emphasizing the importance of immune system activity in regulating the brain’s environment.

## 3. Dysregulation in General Anxiety Disorder and Depression

Emerging research highlights the significant role of inflammation in psychiatric disorders, particularly anxiety and depression. While traditionally understood through cognitive and neurochemical frameworks, increasing evidence suggests that immune dysregulation contributes to both the onset and progression of these conditions. The intricate interplay between the immune system and the central nervous system (CNS) influences neuroinflammation, neurotransmitter balance, and stress responses, shaping their course. Understanding how inflammatory markers relate to generalized anxiety disorder (GAD) and depression can provide valuable insights into the biological mechanisms underlying these disorders.

### 3.1. General Anxiety Disorder

The diagnostic criteria for generalized anxiety disorder (GAD) require excessive worry about a range of issues for at least six months. Individuals find it difficult to control this worry, leading to clinically significant distress or impairment in functioning. The worry is accompanied by at least three of the following symptoms: difficulty concentrating, restlessness, irritability, muscle tension, loss of energy, and sleep disturbances [26].

GAD affects 1.9–5.1% of the population, with 8% of primary care patients diagnosed with the disorder [27]. Studies have also reported anxiety-mediated effects on inflammatory activity in individuals with GAD, though whether this inflammatory response is consistent across all GAD patients remains uncertain [28].

A 2023 meta-analysis found changes in immune cells and inflammatory mediators in GAD patients without other inflammatory conditions. Specifically, GAD patients had higher amounts of basophils, CD45RA+ cells, and CD62L+ cells compared to matched controls. CD45RA+ cells are naive T-cells that, when activated, differentiate into TH1 or TH2 helper T-cells, producing inflammatory cytokines like TNF-α, IL-2, and interferon-gamma (IFN-γ) [29].

Another study observed increased levels of C-reactive protein (CRP), particularly in individuals with late-onset GAD (age 50+) [30]. CRP is a nonspecific acute-phase protein produced in response to inflammation, and it raises the question of whether chronically high CRP levels due to psychological and physical stress might contribute to late-onset GAD [31]. Some studies have also linked high CRP levels with anxiety, particularly in men, suggesting that hormonal differences may influence both inflammation and anxiety [32].

Cell cultures of activated T-cells from GAD patients have shown increased levels of pro-inflammatory cytokines, such as TNF-α and IL-17, and decreased levels of the anti-inflammatory cytokine IL-4. However, the relationship between pro- and anti-inflammatory cytokines in GAD remains unclear, as IL-2—a pro-inflammatory cytokine—was also found to be reduced [33].

In general, the studies point to a trend of increased pro-inflammatory markers and decreased anti-inflammatory markers in GAD patients. However, most of these studies focused on late-onset GAD, which occurs in older individuals and is associated with aging-related inflammation. Advanced age is associated with increased levels of IL-1 and TNF-α compared to younger populations. This is driven by immunosenescence—the decline in immune system function that makes older adults more susceptible to chronic, low-grade inflammation [34]. This raises the question of whether the observed inflammatory markers are due to GAD itself or are simply a consequence of aging. Future studies in younger populations are needed to fully evaluate these trends.

### 3.2. Depression

Depression is strongly linked to an elevated inflammatory profile and the dysregulation of immune and neurotransmitter systems. The relationship between the symptoms of depression and immune signaling is likely bidirectional, with both contributing to the onset and progression of the disorder. This interplay is complex and varies depending on the type, duration, and severity of depressive symptoms [35]. Evidence exists for both altered immune responses to systemic inflammation and the impact of psychological stress on immune function.

Two predominant theories have been used to explain depression’s pathophysiology: the monoamine theory and the stress-induced dysfunction of the hypothalamic–pituitary–adrenal (HPA) axis. The monoamine hypothesis posits that changes in the neurotransmitter and chemical balance in the brain lead to altered neuronal activity, while the second theory highlights the stress-induced imbalance in the HPA axis, resulting in immune dysregulation and inflammation [36,37].

The monoamine hypothesis, developed in the 1950s, was inspired by the discovery that hypertensive patients treated with reserpine, a vesicular monoamine transporter (VMAT) inhibitor which shunts monoamines towards degradation, developed depressive-like symptoms [38,39]. Although this theory remains influential, it does not fully explain all aspects of depression. For instance, treatments such as lithium, which do not directly alter monoamine levels, remain effective for managing depression. In contrast, substances like cocaine, which directly modulate monoamine levels, do not have antidepressant effects, suggesting that additional factors contribute to depression’s onset [36].

In response to psychological stress, the hypothalamus releases corticotropin-releasing hormone (CRH), which triggers pituitary signaling to release adrenocorticotropic hormone (ACTH), ultimately resulting in glucocorticoid release from the adrenal glands. Both acute and chronic stress can alter the sensitivity of the HPA axis, which subsequently affects immune responses [40]. This is thought to occur through dampened monocyte responses, which impair the negative-feedback mechanisms and result in prolonged glucocorticoid elevation [41]. Additionally, activation of the sympathetic nervous system in response to stress signals can further enhance immune activity, promoting cytokine release and upregulating the immune response genes in a beta-adrenergic receptor-mediated fashion [40]. This stress-induced immune activation feeds back to increase CRH production in the hypothalamus, suggesting a positive-feedback loop between inflammation and stress responses [42].

**Figure 1:**
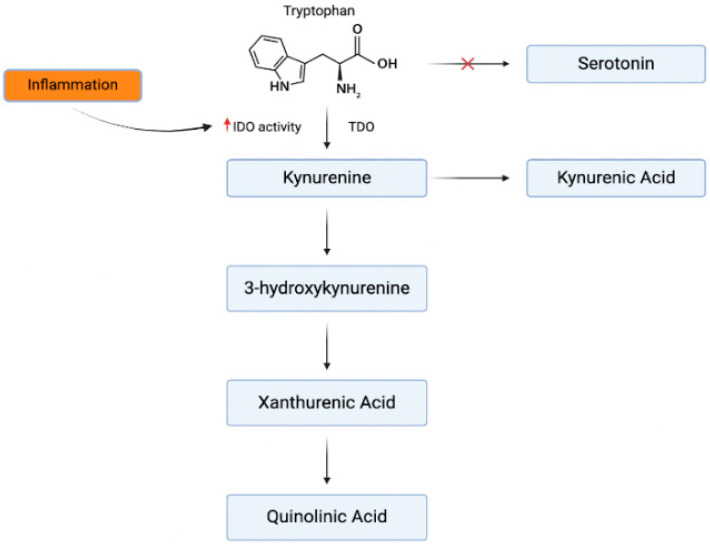
Inflammatory effects on tryptophan metabolism. The kynurenine pathway originates from the same precursor amino acid as serotonin. In times of inflammation, the effects of IDO are amplified and shift tryptophan [43].

Inflammatory responses can also alter the synthesis of serotonin, with tryptophan, the precursor to both serotonin and kynurenine, being diverted toward kynurenine production in inflammatory states. Kynurenine is synthesized via two enzymes, tryptophan 2,3-dioxygenase (TDO) and indoleamine 2,3-dioxygenase (IDO), the latter of which is upregulated by IFN-γ, a pro-inflammatory cytokine (Figure 1) [43]. Elevated kynurenine levels may disrupt serotonin production, and kynurenine metabolites like quinolinic acid can overstimulate N-methyl-D-aspartate (NMDA) receptors, leading to neuronal toxicity and neuroinflammation [44]. This imbalance between kynurenine and serotonin contributes to the neurochemical changes observed in depression.

Preclinical studies using mouse models have provided valuable insights into the effects of depression and stress on brain tissue. One such model utilized repeated social defeat, which mimics long-term exposure to social stressors, reflecting depressive behavior in humans [45]. These studies have shown that chronic stress increases circulating monocytes and pro-inflammatory cytokines, with significant effects on the hippocampus and prefrontal cortex [41]. Furthermore, chronic glucocorticoid exposure in mice upregulates the production of NLRP3 inflammasomes, microglia, and macrophages, implicating stress-induced glucocorticoid elevation in both inflammation and emotional regulation [46].

Inflammatory markers such as IL-1β, IL-6, TNF-α, IL-18, and CRP are commonly observed in animal models of depression. The administration of lipopolysaccharide (LPS) to mice, which induces inflammation, results in behavioral changes that resemble depressive symptoms in humans [47]. Interestingly, IL-1β antagonists can reverse these behavioral changes, supporting the role of inflammation in depression [48].

In humans, heightened inflammation, seen with the administration of exogenous IL-2 or interferon-alpha (IFN-α), has been associated with the onset of depressive symptoms [43]. Retrospective studies have also linked childhood illness and inflammation with an increased risk of depression later in life, although it is difficult to disentangle these effects from the psychological impacts of chronic illness [41]. Additionally, post-mortem analyses of brain tissue from patients with depression have revealed alterations to the markers of microglial, astrocyte, and oligodendrocyte function, including elevated levels of Iba1, a microglial activation marker [49]. Microglial activation has been linked to neuroinflammatory processes, including cerebral ischemia and autoimmune responses within the CNS [50,51].

Given the strong association between inflammation and depression, understanding this relationship on both a molecular and behavioral level has significant clinical implications. Managing inflammation alongside depression may improve treatment outcomes and could offer a new avenue for therapeutic intervention.

Taken together, the inflammatory profiles of GAD and depression suggest overlapping but distinct immunological patterns that reflect the complexity of these disorders. While both conditions show evidence of heightened inflammatory activity, depression is characterized by a more consistent and well-established immune signature, marked by elevated pro-inflammatory cytokines and stress-related neuroimmune changes. In contrast, GAD presents with a more heterogeneous profile, with immune alterations that may be influenced by age, hormonal differences, or comorbid stress-related factors. The variability in GAD’s inflammatory markers, such as mixed cytokine levels and differential CRP expression, raises important questions about whether these changes are intrinsic to the disorder or secondary to other physiological processes like aging or chronic stress. These findings highlight the need for more targeted research, particularly in younger and more diverse GAD populations, to disentangle the contributions of systemic inflammation from those of the disorder itself. Understanding the nuanced immunological distinctions between GAD and depression could ultimately inform more personalized therapeutic strategies, especially as inflammation-targeting treatments continue to emerge.

When comparing neuroinflammatory disorders such as meningoencephalitis and neuritis to psychiatric conditions like anxiety and depression, key distinctions in the nature and drivers of inflammation become evident. Infectious neuroinflammatory conditions are characterized by acute, high-intensity inflammation, with a rapid and well-defined cytokine response aimed at eliminating the pathogens or repairing tissue damage. This response is typically localized and triggered by a specific, identifiable cause, most often microbial invasion or direct injury [52]. In contrast, anxiety and depression are associated with chronic, low-grade systemic inflammation that unfolds gradually over time and lacks a clear pathogenic source. Instead, it is often driven by prolonged psychological stress and dysregulation of the HPA axis. This persistent inflammatory state is believed to subtly alter brain function through disruptions in neurotransmitter metabolism and modulation of neural circuits involved in emotion and behavior. Moreover, while cytokine profiles of infectious neuroinflammation are well characterized and closely tied to disease mechanisms, the specific roles of cytokines in psychiatric disorders remain incompletely understood, highlighting a key area for ongoing investigation.

## 4. Anti-Inflammatory Effects of Antidepressants

With the rising prevalence of anxiety and depression, especially in the wake of the COVID-19 pandemic, medication use has also increased [53,54]. Medications targeting neurotransmitter systems have distinct primary mechanisms, yet many also exert secondary anti-inflammatory effects, which may contribute to their overall therapeutic efficacy.

### 4.1. SSRIs and SNRIs

Selective serotonin reuptake inhibitors (SSRIs) and serotonin/norepinephrine reuptake inhibitors (SNRIs) are first-line pharmacological treatments for conditions such as major depressive disorder (MDD) and anxiety. SNRIs have also been utilized in the treatment of chronic pain syndromes, including fibromyalgia. Numerous studies have evaluated the interaction of SSRIs and SNRIs with the immune system, both in vivo and in vitro. In general, SSRIs/SNRIs have been shown to reduce pro-inflammatory cytokines such as IL-1, IL-6, TNF-α, and IFN-γ, while increasing the anti-inflammatory cytokine IL-10. Specifically, fluoxetine has been found to upregulate IκB, a negative regulator of the NF-κB pathway, in an ischemic brain injury model [55].

A study investigating the treatment of MDD with escitalopram found alterations in the kynurenine metabolism pathway, which is implicated in serotonin regulation. MDD patients treated with escitalopram had a higher ratio of kynurenic acid (an anti-inflammatory metabolite) to quinolinic acid (a pro-inflammatory metabolite), as well as an elevated tryptophan-to-quinolinic acid ratio [56]. Additionally, both sertraline and escitalopram resulted in reduced levels of IL-1β, IL-6, IL-8, IFN-γ, and CRP in patients with GAD [57]. However, there are significant limitations to the meta-analyses that have evaluated the anti-inflammatory effects of SSRIs and SNRIs in patients. These include variability in the baseline inflammatory profiles, the severity and duration of the disease, and individual clinical responses to treatment.

### 4.2. MAOIs

Monoamine oxidase inhibitors (MAOIs) are commonly prescribed for the treatment of various conditions, including MDD, panic disorder, and social anxiety disorder. Monoamine oxidases catalyze the deamination of neurotransmitters such as epinephrine, dopamine, norepinephrine, and serotonin, as well as other amines, including tyramine. The net effect of MAOIs is an increase in the concentration of these substrates in the synaptic cleft, many of which are typically deficient in depression. The positive effects stemming from this mechanism include mood regulation and functional improvements in memory, alertness, arousal, and attention. However, potential adverse effects include orthostatic hypotension, dry mouth, insomnia, diarrhea, and constipation. Additionally, MAOIs are associated with significant drug and food interactions with other serotonergic agents and tyramine-rich foods, posing risks of serotonin syndrome and hypertensive crisis [58].

Beyond the well-known mechanism of action, there is emerging evidence that MAOIs exert additional anti-inflammatory effects, which could play a role in the treatment of MDD. In a murine LPS-induced depression model, the MAOI tranylcypromine was shown to reduce the expression of pro-inflammatory cytokines IL-1β, IL-6, and TNF-α. It also prevented the LPS-induced reduction in the cAMP response element-binding (CREB) protein, thereby increasing the negative regulation of NF-κB gene expression [59].

In another study, investigators administered moclobemide, a reversible MAOI, to healthy individuals. They found that the drug significantly suppressed the unstimulated production of TNF-α and IL-8, while enhancing the production of IL-10, an anti-inflammatory cytokine [60]. It is important to note that while these studies demonstrated significant reductions in inflammation associated with MDD, they could not evaluate whether these changes in inflammation would lead to improvements in the clinical symptoms.

In 1983, a case was reported involving a patient treated with phenelzine for depression, who showed rapid improvement in both her depression symptoms and inflammatory bowel disease (IBD). Her IBD remained stable while on phenelzine but reactivated once she discontinued the medication [61]. Further, MAOIs may reduce metabolic end products like hydrogen peroxide, thereby decreasing oxidative stress, which is involved in inflammatory cell signaling [62]. This suggests a potential secondary mechanism for their use in treating psychological disorders.

### 4.3. NDRI

Bupropion is commonly prescribed for the treatment of MDD and smoking cessation. It acts through the weak inhibition of the reuptake of dopamine and norepinephrine (NDRI) in the brain, thereby increasing the synaptic availability of these neurotransmitters. Common adverse effects include tachycardia, weight loss, rhinitis, tremors, dry mouth, a lowered seizure threshold, and the potential worsening of suicidal ideation, particularly in children and young adults.

In addition to its primary action on neurotransmitter systems, bupropion has demonstrated anti-inflammatory effects. Studies in patients with depression and concurrent conditions such as psoriasis, Crohn’s disease, or fibromyalgia have shown that bupropion use is associated with decreased inflammatory markers compared to the control groups. Notably, the levels of pro-inflammatory cytokines like IL-6, GM-CSF, and IL-12p40 were significantly suppressed, especially at higher bupropion doses. It has been proposed that bupropion reduces the phosphorylation of p38, a kinase responsible for the differentiation of pro-inflammatory macrophages [63]. Supporting this claim, additional studies have shown that bupropion can suppress TNF-α and IFN-γ production in both animal models and humans [64,65]. Other studies have suggested that bupropion leads to a significant increase in anti-inflammatory cytokines, such as IL-4, IL-5, IL-10, and IL-13, in MDD patients after 4 weeks of treatment [66].

However, there is no consensus regarding the optimal dosage that produces these anti-inflammatory effects, as variability exists between the studies. Additionally, the relationship between the improvement of inflammatory diseases (e.g., Crohn’s disease or fibromyalgia) and the modulation of cytokine levels by bupropion remains unclear. For instance, it was not always specified when the patients in these studies stopped taking other anti-inflammatory medications (e.g., sulfasalazine for Crohn’s disease), which may have influenced the observed effects. Nevertheless, one study that evaluated bupropion monotherapy over 12 weeks reported a significant decrease in TNF-α levels in patients who subjectively experienced a reduction in their MDD symptoms. Specifically, TNF-α levels decreased from 4.45 pg/mL to 2.11 pg/mL, with this change corroborated by a reduction in the Hamilton Depression Rating score compared to matched controls [67].

### 4.4. Tricyclic Antidepressants

Tricyclic antidepressants (TCAs) operate through several mechanisms. The primary action is the inhibition of serotonin and norepinephrine reuptake; however, they also exhibit some degree of inhibition of alpha-adrenergic, muscarinic, and histamine receptors [68]. This class of medication is now considered a common second-line treatment for depression due to its narrow therapeutic window. In addition to their use in depression management, TCAs have proven effective in treating certain neuropathic pain disorders. A 2015 meta-analysis found that amitriptyline demonstrated positive outcomes in pain management when compared to a placebo [69].

TCAs have also been observed to possess anti-inflammatory properties. Preclinical studies using microglial cells have linked certain TCAs with reductions in cytokines and inflammatory mediators in response to LPS-induced inflammation [70]. The potential mechanisms for these effects include the activation of the PI3K/Akt signaling pathway and the attenuation of NLRP3 inflammasome-induced inflammation [71,72]. While these effects have primarily been studied in mouse models, similar anti-inflammatory effects have been observed in human tissues. One study evaluating the response of LPS-stimulated whole human blood found that clomipramine administration led to a decrease in IFN-γ levels, along with an increase in IL-10 [73]. These findings were further supported by an in vivo study which showed that amitriptyline treatment for depression resulted in a reduction in TNF-α activity [74].

When considering the broader context, it appears that the modulation of inflammation plays a significant role in the symptomatic management of depression with TCAs. Although the wide range of adverse effects associated with TCAs makes them less desirable as a first-line treatment compared to other medications, such as SSRIs and SNRIs, their association with specific immune pathways may explain their effectiveness in patients who are refractory to first-line therapies. If the relationship between TCAs and cytokine modulation is indeed a key factor in their efficacy, it would be worthwhile to explore adjunct therapies with similar anti-inflammatory effects but fewer side effects, which could benefit patients starting on first-line treatments.

## 5. Non-Prescription Medicine

Given the association between inflammation and the immune response in psychiatric illness, it is reasonable that therapies with anti-inflammatory properties, such as nonsteroidal anti-inflammatory drugs, corticosteroids, cytokine inhibitors, and other supplements, have been explored for the management of depression and anxiety. In theory, by reducing the generation of inflammatory mediators and signals, these treatments may help mitigate the dysregulation of immune cell activity and regulate neurotransmitter responses.

Many of the anti-inflammatory medications that have been assessed in studies are prescription-based and come with potentially significant side effects. For example, corticosteroids are anti-inflammatory medications that target gene expression through interactions with local glucocorticoid receptors [75]. Similarly, cytokine inhibitors target specific pro-inflammatory mediators or the signals that initiate their production [76]. However, the long-term use of glucocorticoids is generally avoided due to their extensive side-effect profile. A similar caution is applied to cytokine inhibitors, which are associated with immunosuppression and other potential adverse effects.

For these reasons, the remainder of this discussion focuses on several classes of over-the-counter medications and supplements that have a less significant side-effect profile, yet may still offer potential benefits in managing inflammation and warrant further investigation.

### 5.1. Nonsteroidal Anti-Inflammatory Drugs

Nonsteroidal anti-inflammatory drugs (NSAIDs) work by blocking the cyclooxygenase (COX) enzymes, COX-1 and COX-2. These enzymes are responsible for generating pro-inflammatory compounds, such as prostaglandins, leukotrienes, prostacyclin, and thromboxane, which play key roles in inflammatory signaling [77]. NSAIDs are widely used and generally well tolerated, often considered a first-line treatment for managing both acute and chronic inflammation.

Given their favorable tolerability, NSAIDs have been explored as potential treatments for inflammation associated with psychiatric diseases. NSAIDs’ anti-inflammatory effects in the brain are thought to reduce microglial activation and attenuate the release of pro-inflammatory cytokines, such as IL-6 and TNF-α, in the CNS. This reduction in inflammatory signaling could potentially result in more regulated immune cell activity in the brain. Additionally, by reducing the CNS’s inflammatory state, it is theorized that NSAIDs may help restore the balance of neurotransmitter levels, thus influencing neuronal signaling.

Numerous studies and meta-analyses have investigated the impact of NSAIDs on inflammatory markers and their potential correlation with psychiatric symptoms, particularly depression. The findings have been mixed, with some studies suggesting benefits while others have reported no clinically significant effects [78,79,80]. An early review by Köhler et al., which compared NSAID use to direct cytokine inhibitors in patients with depression or depressive-like symptoms, found a statistical significance favoring NSAIDs over placebos. This effect was most pronounced in patients with elevated CRP and IL-6 levels. Notably, the COX-2 selective inhibitor celecoxib showed the most promise, providing benefits both as an adjunct therapy and monotherapy [80]. These effects could be attributed to the modulation of neuro-immune signaling and subsequent changes in neuronal activity. However, this hypothesis has not been directly tested to date.

It is important to note that the findings of this review have been critiqued due to the heterogeneity among the study participants and study designs. Recent analyses have also pointed out that small sample sizes and potential biases may have influenced the conclusions drawn from the original meta-analysis [81]. Additionally, a population-based study in Denmark indicated that the specific NSAID and duration of NSAID use could impact their efficacy in treating depressive symptoms. These investigators found that the continued use of low-dose aspirin was associated with a lower incidence of depression, while high-dose aspirin and non-aspirin NSAIDs were associated with a higher incidence. It has been hypothesized that prolonged NSAID use may disrupt normal immune activity or alter neurotransmitter signaling, potentially affecting the kynurenine pathway [82].

A more recent review of anti-inflammatory treatments considered the growing body of research and the critiques of earlier studies. This review found that anti-inflammatory agents showed promise as adjunct therapies to antidepressants in patients with a heightened inflammatory state [78,83].

Given the inconsistent findings on NSAID use for managing depression and anxiety, it remains difficult to draw a definitive conclusion about their therapeutic value for psychiatric conditions. The mechanisms by which NSAIDs impact brain activity and psychiatric symptoms are not well understood and require further investigation. Despite their potential to target components of the inflammatory response, this area of research demands more rigorous and larger-scale randomized controlled trials before any definitive conclusions can be made. Future studies should focus on constructing well-defined cohorts that minimize comorbidities, avoid bias, and emphasize the inflammatory component of depression to reduce heterogeneity.

### 5.2. Palmitoylethanolamide

**Figure 2:**
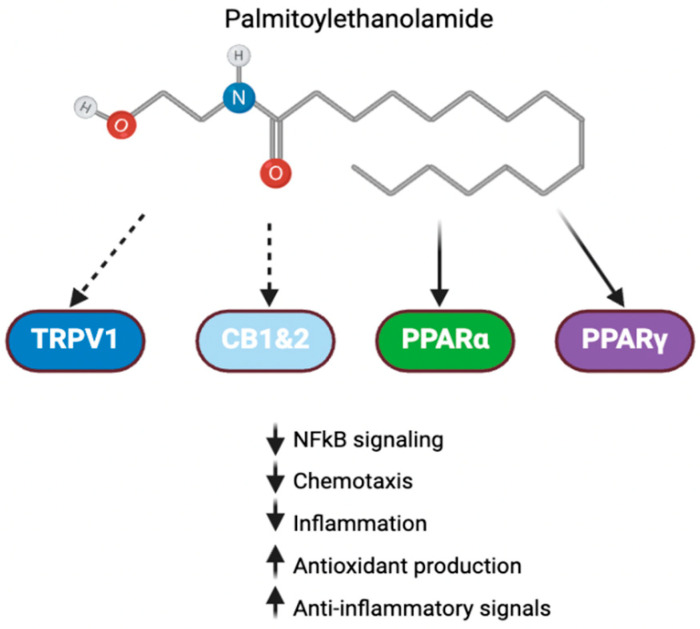
PEA signaling in inflammation. Palmitoylethanolamide interacts with a number of receptors involved in inflammation. Direct activity with PPAR and indirect activity with cannabinoid receptors and TRPV1 allow it to exert anti-inflammatory effects [84].

Palmitoylethanolamide (PEA) is an endogenous lipid-like mediator found in plants, milks, and seeds. Its anti-inflammatory mechanisms are thought to be linked to its interaction with the peroxisome proliferator-activated receptor (PPAR) signaling pathway, specifically the alpha and gamma subtypes (Figure 2) [84]. These pathways are typically activated in response to oxidative damage and lipid peroxidation, though stimulatory ligands can also be administered via dietary supplementation or parenterally.

The PPAR pathways exert their effects by initiating transcriptional changes within cells. PPARs typically form a complex with retinoid X receptors (RXRs), which bind to gene promoter motifs to influence gene expression [85]. The downstream effects of PPAR activation are broad and include reducing inflammation and cell death through interactions with NF-κB, AP-1, JAK/STAT signaling, scavenging reactive oxygen species (ROS), and promoting lipid catabolism.

**Figure 3:**
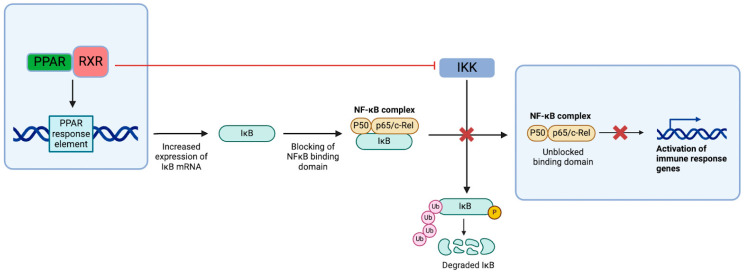
PPAR-dependent NF-κB pathway suppression. In addition to the direct inhibition of IKK-induced activation of NF-κB, PPAR complexes with RXR, which allows for interfacing with the response elements in the nucleus and upregulates the transcription of IkB mRNA. The increase in IkB results in the blocking of active regions in the NF-κB complex and preventing pro-inflammatory signaling [86].

The PPAR-RXR complex suppresses NF-κB signaling by binding to the PPAR response element (PPRE) in the IkB-α gene, a negative regulator of NF-κB signaling. The complex also inhibits IKK, which normally phosphorylates IkB, leading to its ubiquitination (Figure 3) [86]. Additionally, PPAR-α has been shown to reduce the chemotaxis of inflammatory cells by lowering levels of leukotriene B4 (LTB4). LTB4 itself activates PPAR, creating a negative-feedback loop that helps regulate inflammatory responses. This mechanism has been demonstrated in studies with PPAR-α knockout mice, which experienced excessive inflammation due to LTB4/5-LOX activation, a process that was attenuated with PPAR overexpression in transfected cells [87].

PEA is also thought to reduce inflammation by managing ROS through increases in the expression of antioxidant enzymes, such as catalase, heme oxygenase-1, Mn-superoxide dismutase, and CuZn-superoxide dismutase [86]. These changes occur at the transcriptional level, leading to the increased production of these scavenger compounds [88].

PEA has primarily been studied for its potential in managing chronic pain due to its anti-inflammatory properties [89]. These effects have also sparked interest in its potential use as an adjunct treatment for psychiatric conditions. A recent human trial of PEA as an adjunct to citalopram demonstrated a significant reduction in the Hamilton Depression Rating Scale (HAM-D) scores, particularly in male patients, compared to citalopram alone. This effect may be partly due to PEA’s interaction with both PPAR and transient receptor potential vanilloid-1 (TRPV1) pathways [90].

The effects of TRPV1 are complex, with both pro-inflammatory and anti-inflammatory activities. TRPV1 activation is associated with inflammatory processes, thermoception, and nociception [91]. TRPV1’s pro-inflammatory activity is due to the release of cytokines and chemokines, such as IL-1β, IL-4, IL-16, and CXCL, among other factors like endothelin [92]. These mediators can further sensitize the TRPV1 pathway [93]. However, TRPV1 also has anti-inflammatory properties, such as desensitizing its activity through repeated stimulation, which is thought to explain the analgesic properties of capsaicin, a well-known TRPV1 activator [94]. PEA, a partial TRPV1 agonist, may inhibit inflammatory signaling by interacting with this receptor [95]. The complete mechanism by which TRPV1 regulates inflammation is not yet fully understood, and further research is needed.

A third proposed mechanism by which PEA modulates inflammatory signaling is through the indirect augmentation of endocannabinoid signaling. This is believed to activate cannabinoid receptors [96]. Initially, it was thought that PEA’s effect was due to the inhibition of fatty acid amide hydrolase (FAAH), the enzyme responsible for the degradation of endocannabinoids. However, recent studies have suggest that PEA may reduce FAAH expression, rather than inhibiting its activity directly [96,97]. These promising findings warrant further exploration to better understand the potential of PEA in targeting inflammation and its connection to psychiatric illness.

With its anti-inflammatory effects, it is plausible that PEA could influence psychiatric symptoms and treatment responses in a manner similar to that of other anti-inflammatory therapies. By reducing inflammation, PEA could help regulate the dysregulated kynurenine pathway and alter immune cell activity in the central nervous system, providing potential benefits either directly or as an adjunctive therapy.

Given its potential, clinical interest in PEA has been steadily increasing. Recent meta-analyses and systematic reviews have suggested that PEA is effective in managing inflammatory and chronic pain conditions, showing general tolerability, minimal side effects, and positive trends in reducing pain and inflammation compared to placebo [98]. However, it is important to note that a 2022 investigation by Scuteri et al. highlighted the variability in the secondary outcomes across the trials, suggesting that differences in study designs make a definitive meta-analysis challenging [99]. While more studies are needed to strengthen the evidence base, PEA holds promise for clinical use in managing conditions where inflammation is a key factor, including psychiatric disorders with immune dysregulation, such as depression and anxiety.

### 5.3. Omega-3

Omega-3 fatty acids (FAs) are polyunsaturated fats that play a crucial role in various physiological functions. Since the body cannot synthesize them, individuals must obtain omega-3 FAs through their diet or supplements. Two key omega-3 FAs are eicosapentaenoic acid (EPA) and docosahexaenoic acid (DHA), both commonly found in fish [100].

**Figure 4:**
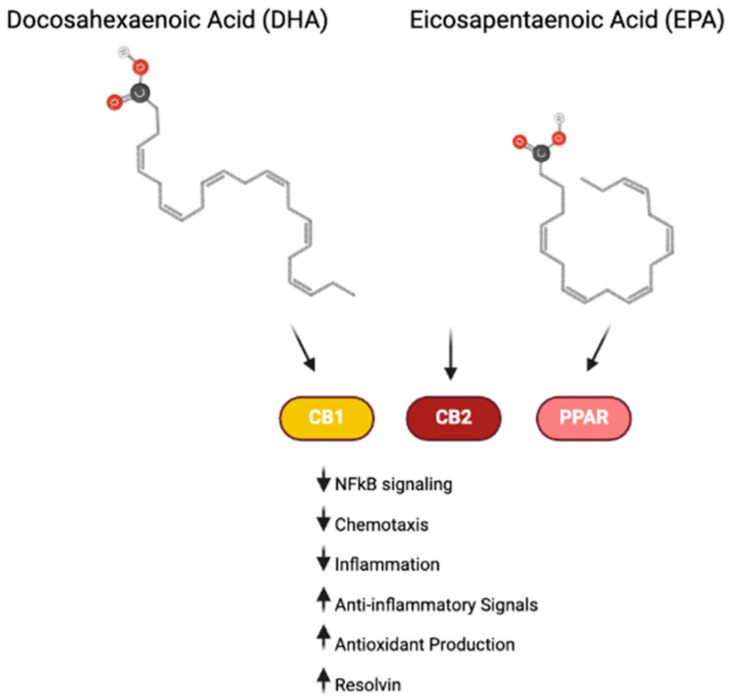
Omega-3 signaling in inflammation. Omega-3 compounds DHA and EPA and their downstream endocannabinoid products interact with cannabinoid receptors and PPAR to exert anti-inflammatory effects [101,102].

Endocannabinoids (eCBs), such as eicosapentanoyl ethanolamide (EPA-EA) and N-docosahexaenoylethanolamide (DHA-EA), are derived from omega-3 FAs, including DHA and EPA. The consumption of omega-3 FAs promotes the production of these endocannabinoid-like molecules. These omega-3-derived eCBs can exert their effects through both cannabinoid-dependent and -independent pathways. For instance, they can target CB1 and CB2 receptors, where they exhibit varying degrees of affinity and agonistic activity (Figure 4). These receptors, present on immune cells, play a key role in modulating the immune system and inflammatory pathways, both peripherally and within the brain [101]. Moreover, omega-3 FAs and their derived eCBs can also act directly on inflammatory cells via the PPAR signaling pathway to reduce inflammation and manage inflammatory debris [102].

Through these mechanisms, omega-3 FAs and their derived eCBs have demonstrated anti-inflammatory properties. Research has shown that EPA-EA can lead to a dose-dependent reduction in IL-6 and an increase in IL-10 [103]. Additionally, omega-3 FAs have been reported to lower levels of TNF-α, a key inflammatory mediator [104].

Omega-3 FAs also influence inflammation by altering cell membranes. Inflammatory cells are often enriched with the omega-6 fatty acid arachidonic acid, which is metabolized to produce eicosanoids, such as prostaglandins and leukotrienes [105]. By administering EPA and DHA, the composition of these cell membranes can be modified, leading to a reduction in pro-inflammatory eicosanoids. Furthermore, EPA and DHA can give rise to resolvins, a class of molecules that help resolve inflammation by stimulating macrophages and counteracting pro-inflammatory molecules [106]. These changes in the metabolism of arachidonic acid and the production of resolvins underscore the anti-inflammatory benefits of EPA and DHA.

Given these anti-inflammatory effects, omega-3 FAs and their derived eCBs present a promising therapeutic avenue for mitigating chronic inflammation. This makes them a potentially valuable tool for reducing inflammation associated with psychiatric disorders, potentially improving clinical symptoms related to these conditions.

### 5.4. Cannabidiol

Phytocannabinoids are plant-derived molecules that can act on the endocannabinoid system. The relevant molecules in this class include Δ^8^-tetrahydrocannabinol (THC), Δ^9^-THC, and cannabidiol (CBD). The former two exert psychoactive effects while CBD does not. Despite this minor difference, all three have been found to have anti-inflammatory and antioxidant effects, with CBD being the most widely accessible as an over-the-counter product throughout most of the United States in a variety of creams, inhalants, and ingestible forms. The prominent mechanisms underlying its anti-inflammatory activity involve activation of the PPAR system, as previously discussed in this review, along with additional reductions in inflammatory interleukins, TNF-α, T-cell activity, and cellular migration [107]. CBD has also been shown to increase the levels of endogenous endocannabinoids, including 2-arachidonoylglycerol (2-AG), which possess known anti-inflammatory effects. 2-AG has been reported to reduce neuroinflammation in the post-ischemic brain by decreasing BBB permeability and inhibition of pro-inflammatory cytokine signaling [108]. CBD exhibits antioxidant effects through both direct and indirect mechanisms involving redox activity. Directly, CBD can neutralize free radicals and mitigate the Fenton reaction by chelating transition metals. Indirectly, CBD is believed to act at the transcriptional level, with observed increases in mRNA expression for superoxide dismutase enzymes. Additional indirect effects include a reduction in lipid peroxidation and subsequent downstream pro-oxidative signaling typically associated with oxidative stress [107]. While research in this area is promising, further studies are necessary to definitively establish clinical efficacy. Several phase 1 and phase 2 trials investigating CBD, THC, or their combination in chronic inflammatory diseases have reported favorable outcomes [109].

### 5.5. N-Acetylcysteine

N-Acetylcysteine (NAC) is a widely available over-the-counter medication and dietary supplement known primarily for its mucolytic properties and its use in treating acetaminophen overdose [110]. Recently, there has been growing interest in expanding the use of NAC for managing psychiatric disorders, particularly for enhancing the effectiveness of antidepressant treatments. The potential therapeutic effects of NAC in this context are thought to stem from its ability to mitigate oxidative stress and regulate neurotransmitter dysregulation, both of which are linked to neuroinflammation commonly observed in psychiatric conditions.

In the body, NAC primarily functions as an antioxidant. It acts as a precursor to glutathione (GSH), a potent endogenous antioxidant that helps neutralize ROS. NAC facilitates the synthesis of GSH by donating a cysteine group, which is essential for the formation of GSH. Once synthesized, GSH contains two thiol groups that enable it to reduce free radicals, thereby preventing oxidative damage to cellular structures [110]. Chronic oxidative stress can lead to disruptions in normal brain function, including impairments in mitochondrial activity and an imbalance between oxidative and antioxidative forces.

Beyond its antioxidant properties, NAC has been shown to influence inflammatory pathways. Studies have suggested that NAC can reduce the transcription of pro-inflammatory mediators, such as NF-κB, and lower levels of common inflammatory cytokines, like IL-1β, IL-6, TNF-α, and IFN-γ [111]. Notably, research on oral NAC supplementation has indicated that it primarily affects markers of inflammation, such as CRP and IL-6 [112]. As discussed earlier, oxidative stress can contribute to the disruption of the BBB and interfere with brain function. By increasing the synthesis of antioxidative GSH, NAC helps the body counteract the harmful effects of oxidative stress, potentially reducing the inflammatory damage seen in psychiatric conditions. Another proposed mechanism for NAC’s effects on psychiatric disorders is its role in regulating glutamate neurotransmission. NAC may help balance glutamate levels at the synapse and modulate ligand binding at glutamate receptors, which plays a critical role in neural communication and plasticity [111].

Despite its promising mechanisms, clinical studies on NAC’s effectiveness in treating psychiatric disorders remain limited, and the results thus far have been mixed. However, many of the available studies have suggested favorable outcomes, particularly for enhancing the response to antidepressant therapy. Further controlled studies are necessary to fully understand the potential role of NAC in managing psychiatric conditions and its long-term benefits.

## 6. Non-Pharmaceutical Interventions

While pharmaceutical therapies are commonly used to treat depression and anxiety, various non-pharmacological interventions are also widely employed to complement or enhance treatment outcomes. These approaches include therapies that target the psychosocial aspects of these conditions, as well as those that alter neuronal firing patterns using electrical currents or magnetic waves.

### 6.1. Psychosocial Therapies

Psychosocial interventions, particularly cognitive behavioral therapy (CBT), have demonstrated considerable success and become integral components of psychiatric treatment regimens. CBT is a collaborative process between the therapist and patient aimed at identifying and altering dysfunctional thought and behavior patterns to improve mood and overall well-being [113]. It has been shown to provide benefits both as a standalone therapy and as an adjunct to medication. A 2020 meta-analysis of various psychosocial therapies identified CBT—especially when combined with other therapeutic methods—as the most effective for eliciting changes to immune markers and cell counts [114]. Specifically, inflammatory markers, such as TNF-α, IL-6, CRP, MCP4, and ICAM, were observed to decrease with CBT. Interestingly, IL-10 levels increased notably when CBT was administered without pharmacological therapy, suggesting a potential benefit of psychosocial therapies on immune regulation. Moreover, a higher baseline inflammation was associated with reduced symptomatic improvement from psychological interventions [115].

### 6.2. Mindfulness-Based Techniques

Another popular approach for managing depression and anxiety is mindfulness-based interventions. These therapies emphasize grounding one’s attention and fostering acceptance of one’s experiences [116]. Several randomized trials have shown that mindfulness techniques, while generally less robust than CBT, can result in mild reductions in inflammation, particularly in CRP levels. However, these techniques have not been shown to outperform CBT in reducing inflammatory markers [117]. Similarly, while yoga—often coupled with mindfulness practices—has been linked to reductions in IL-6 in some studies, a review of 42 studies could not definitively attribute these changes to yoga itself, as the effects may have stemmed from the meditative mindfulness aspects rather than the physical practice [118]. Without clearer evidence, it is premature to recommend yoga as an add-on therapy for targeting inflammation in psychiatric conditions.

Despite the promising results of psychosocial therapies, their effect on inflammation may be limited by several factors. Studies have suggested that higher inflammation levels at baseline can reduce the effectiveness of these therapies. Furthermore, challenges exist in standardizing patient cohorts, as regulating psychological states during therapy is not feasible. Future studies that stratify responders based on inflammatory levels could provide more meaningful insights into the link between psychotherapy and inflammation. More robust trials with larger sample sizes and consistent methodology are needed to fully evaluate the potential of non-pharmacological therapies for managing psychiatric symptoms and their associated inflammatory effects.

### 6.3. Neuronal Signaling and Brain Stimulation Therapies

In addition to psychosocial approaches, electroconvulsive therapy (ECT) and transcranial magnetic stimulation (TMS) represent interventions that directly target neuronal signaling and brain activity. ECT involves the use of electrical currents to induce brief seizure activity in the brain, typically over multiple sessions. Though the mechanism remains unclear, ECT has been shown to temporarily increase inflammation, followed by enhanced pro-neurogenesis signaling and a reduction in inflammatory markers. There is also some evidence from animal studies suggesting that ECT increases microglial activity, followed by a subsequent decrease in microglial markers [119]. While the mechanisms of action of ECT warrant further exploration, the therapy has demonstrated efficacy for severe cases of depression.

Transcranial magnetic stimulation (TMS), on the other hand, uses magnetic fields to modulate neuronal activity, with research indicating that it influences action potentials, neurotransmitter release, calcium signaling, and glial activity [120]. Recent studies have suggested that TMS may reduce HPA axis activity and inflammatory mediators, such as IL-1β, IL-6, and TNF-α [121]. While the mechanisms of TMS are still being investigated, it holds promise for managing depression, particularly in patients with treatment-resistant forms.

Overall, non-medication management techniques for MDD and GAD have shown promise for reducing the inflammatory burden associated with these conditions. While CBT and mindfulness-based therapies provide some benefit for managing symptoms and modulating inflammation, the research is less comprehensive compared to that on pharmacological interventions. ECT and TMS are generally reserved for more severe or refractory cases, but the evidence for their efficacy in modulating brain inflammation and neuronal activity is growing. As these therapies expand, further research into their long-term effects on brain inflammation, cellular activity, and neurotransmitter signaling will be essential to better understand their potential role in managing psychiatric disorders. A summary of the general trends of immune response to the treatments reviewed is illustrated in Table 3.

## 7. Limitations

Several limitations must be acknowledged in this review. The complexity of the interactions between neuro-immune and neuro-endocrine signaling remains incompletely understood. These interconnected systems are not fully characterized, and their precise interactions are still being explored. Experimental models—whether in-vitro, animal, or human—each offer valuable insights but also present inherent differences. These discrepancies can lead to variations in how these systems function across species and experimental settings, potentially influencing the generalizability of the findings.

Another significant limitation is the lack of uniformity across clinical trials focused on the inflammatory components of psychiatric disorders and their management. A substantial number of studies have varied in several key aspects, including their sample size, study duration, efficacy measures, and the characteristics of the study populations. These inconsistencies have contributed to a broad range of results, making systematic reviews and meta-analyses more challenging to interpret. As a result, drawing definitive conclusions regarding the most effective treatment or adjunctive therapy is difficult without a clearer consensus from well-standardized trials.

To improve the quality of evidence, large-scale studies with more homogenous cohorts—defined by factors such as inflammatory levels, disease duration, and symptom type—would be invaluable. Such studies could help eliminate potential biases, provide clearer insights into treatment efficacy, and contribute significantly to understanding the relationship between inflammation and psychiatric symptomatology. Additionally, further clinical research into the underlying mechanisms of psychiatric illnesses may help bridge existing knowledge gaps, strengthening the connections between specific symptoms and their inflammatory causes.

## 8. Conclusions and Future Directions

Mental health conditions, such as anxiety and depression, are prevalent across all age groups and communities [122,123]. The World Health Organization has noted significant increases in these conditions, particularly following the COVID-19 pandemic. Given their widespread impact, it is crucial to deepen our understanding of these disorders. Currently, it is widely recognized that these conditions are multifaceted, and are often linked to the dysregulation of various systems, including inflammation, neuronal activity, neurotransmitter balance, and immune cell function.

Beyond advancing our knowledge of the mechanisms underlying these disorders, it is also vital to explore how existing treatments influence these interconnected systems. A better understanding of the pathophysiology behind the development and treatment of anxiety and depression could pave the way for novel prevention and management strategies. For instance, exploring how inflammation affects brain function and how neurotransmitters influence immune cell activity may provide valuable insights into disease progression and symptom manifestation. Additionally, investigating how stress impacts cellular signaling and behavior could reveal the potential bidirectional relationship between immune function and stress in the development of psychiatric symptoms.

Furthermore, more research is needed to examine the effects of current medications on inflammation and immune signaling pathways. This would help clarify how pharmacological and non-pharmacological interventions modulate these processes. Rigorous, large-scale studies are essential for advancing our understanding of these mechanisms, which may ultimately lead to more individualized and effective treatment strategies for patients.

## Figures and Tables

**Table 1:** Immune system mediators. Immune system mediators are involved in both pro- and anti-inflammatory processes [13].

Immune System Mediators		
	Classification	Key Functions in Immune Response and Inflammation
IL-1β	Pro-inflammatory Cytokine	Promotes fever, leukocyte activation, and acute inflammation; involved in neuroinflammation and chronic disease progression.
IL-2	Pro-inflammatory Cytokine	Stimulates T-cell proliferation, enhances immune response, and supports regulatory T-cell function.
IL-4	Anti-inflammatory Cytokine	Induces Th2 cell differentiation, promotes antibody class switching to IgE, and inhibits macrophage activation.
IL-5	Anti-inflammatory Cytokine	Stimulates eosinophil proliferation and activation, playing a key role in allergic inflammation.
IL-6	Pro-inflammatory Cytokine	Mediates acute-phase response, fever, and B-cell activation; can have both pro- and anti- inflammatory effects.
IL-8	Chemokine (CXCL8)	Recruits neutrophils to inflammation sites, promotes angiogenesis, and enhances tissue remodeling.
IL-10	Anti-inflammatory Cytokine	Suppresses pro-inflammatory cytokine production, regulates immune response, and promotes tolerance.
IL-12	Pro-inflammatory Cytokine	Enhances NK- and T-cell cytotoxic activity, promotes Th1 differentiation, and stimulates IFN-γ production.
IL-12p40	Cytokine Subunit	Component of IL-12 and IL-23; regulates Th1 and Th17 immune responses and influences inflammation.
IL-13	Anti-inflammatory Cytokine	Supports Th2 responses, regulates mucus production, and suppresses macrophage pro-inflammatory activity.
IL-16	Chemokine-like Cytokine	Acts as a chemoattractant for CD4+ T-cells, promotes immune cell migration, and regulates inflammation.
IL-17	Pro-inflammatory Cytokine	Drives Th17 responses, promotes neutrophil recruitment, and plays a role in autoimmune diseases.
IL-18	Pro-inflammatory Cytokine	Enhances IFN-γ production, stimulates NK- and T-cell activity, and amplifies inflammatory responses.
TNF-α	Pro-inflammatory Cytokine	Induces fever, apoptosis, and systemic inflammation; plays a major role in chronic inflammatory diseases.
CRP	Acute-phase Protein	Produced in response to IL-6; marker of systemic inflammation and predictor of cardiovascular risk.
TGF-β	Anti-inflammatory Cytokine	Regulates immune tolerance, inhibits pro-inflammatory cytokines, and promotes tissue repair and fibrosis.
IFN-α	Type I Interferon	Antiviral response mediator, enhances immune surveillance, and modulates T-cell activity.
IFN-γ	Type II Interferon	Activates macrophages, promotes Th1 differentiation, and enhances antigen presentation.
NF-κB	Transcription Factor	Regulates inflammatory gene expression, immune cell activation, and responses to stress and infection.

**Table 2:** Microglial response to common neurotransmitters. Microglial response to neurotransmitters is variable based on ligand type, microenvironment, and receptor subtype [20,21,22,23,24].

Microglial Response to Common Neurotransmitters		
Neurotransmitter	Net Effect	Effects
Acetylcholine	Suppressive	Less responsive to IFN-γ; reduction in free radical generation; decreased LPS-induced TNF-α.
Norepinephrine	Suppressive	Reduction in IL-6 and TNF-α; free radical attenuation.
Serotonin	Suppressive	Reduced TNF-α and IL-6 (in vitro).
	Stimulatory	Motility with phagocytosis; NF-κB signaling.
Dopamine	Suppressive	Reduction in NF-κB signaling (D1).
	Stimulatory	Increased NF-κB signaling (D2); release of IL-6 and IL-1β; increased chemotaxis.
Substance P	Stimulatory	Potentiates LPS activity; chemotaxis; microglial activation.

**Table 3:** General trends in immune mediators in response to various treatments. General trends among depression and anxiety treatments show an overall anti-inflammatory pattern [55,57,59,60,63,64,65,66,73,74,80,86,87,92,103,104,107,111,112,115,117,121].

General Trends in Immune Mediators in Response to Various Treatments											
	IL-1	IL-6	TNF-α	IFN-γ	NF-κB	IL-8	CRP	IL-10	IL-4	IL-5	IL-13
SSRI/SNRI	↓	↓	↓	↓	↓ *	↓	↓	↑			
MAO-I	↓	↓	↓		↓ **	↓		↑			
NDRI		↓	↓	↓				↑	↑	↑	↑
TCA			↓	↓				↑			
NSAID		↓	↓								
PEA					↓ ***						
Omega-3		↓	↓					↑			
CBD	↓	↓	↓								
NAC	↓	↓	↓	↓			↓				
CBT		↓	↓				↓	↑			
TMS	↓	↓	↓								

↓: decreased. ↑: increased. * Upregulation of IκB (increased negative regulation). ** Upregulation of cAMP response element-binding protein (increased negative regulation). *** Upregulation of IκB and inhibition of Iκκ (increased negative regulation).

## Data Availability

Data sharing is not applicable to this article as no new data were created or analyzed in this study.
